# Co-existence of extended-spectrum β-lactamases *bla*_CTX-M-9_ and *bla*_CTX-M-15_ genes in *Salmonella* species isolated from febrile and diarrhoeagenic patients in Lagos, Nigeria: a cross-sectional study

**DOI:** 10.1186/s40001-022-00960-0

**Published:** 2023-01-02

**Authors:** Christopher O. Fakorede, Kehinde O. Amisu, Morteza Saki, Kabiru O. Akinyemi

**Affiliations:** 1 Department of Microbiology, Faculty of Science, Lagos State University, Lasu Post Office, Ojo, P.O. Box 0001, Lagos, Nigeria; 2 Department of Microbiology, Faculty of Medicine, Ahvaz Jundishapur University of Medical Sciences, Ahvaz, Iran; 3 Infectious Ophthalmologic Research Center, Imam Khomeini Hospital Clinical Research Development Unit, Ahvaz Jundishapur University of Medical Sciences, Ahvaz, Iran

**Keywords:** Non-typhoidal *Salmonella*, Diarrhoeal, MDR, *Bla*_CTX-M-9_, *Bla*_CTX-M-15_

## Abstract

**Background:**

Resistance to different antimicrobial classes by *Salmonella* species has generated a global public health concern. The spread of extended-spectrum β-lactamases (ESBLs) *bla*_CTX_ gene variants is also increasing. This study aimed to investigate the antibiotic resistance and the carriage of *bla*_CTX-M-9_ and *bla*_CTX-M-15_ as well as the quinolone resistance gene (*qnr*B19) among *Salmonella* species from hospitalised patients in Lagos, Nigeria.

**Methods:**

In this cross-sectional study from April 2021 to August 2021, a total of 508 samples were collected from hospitalised patients. The samples were subjected to standard microbiological investigation. All the isolates were identified using API 20E kits and real-time polymerase chain reaction (RT-PCR). The in vitro antibiotic susceptibility testing (AST) was investigated using the disk diffusion method. Detection of antibiotic resistance and virulence gene makers was conducted using RT-PCR.

**Results:**

In total, 24 *Salmonella* species were identified. All the isolates were non-typhoidal *Salmonella* isolates. None of the isolates screened was *S.* Typhi and *S*. Paratyphi. Most of the isolates were susceptible to imipenem, ciprofloxacin, ofloxacin and gentamycin, while a high level of resistance to all cephalosporins, penicillin, and some carbapenems was observed. In total, 79.2% (19/24) of the *Salmonella* isolates harboured the *bla*_CTX-M_ variant including 54.2% (13/24) *bla*_CTX-M-9_ and 12.5% (3/24) *bla*_CTX-M-15,_ while co-habitation of *bla*_CTX-M-9_ and *bla*_CTX-M-15_ was observed in 12.5% (3/24) of the isolates, respectively. None of the isolates harboured quinolone-resistant *qnr*B19 gene and virulence gene *stn*. However, *inv*A gene was present in 66.7% (16/24) of all isolates.

**Conclusions:**

This study is considered the first report of *bla*_CTX-M-9_ and *bla*_CTX-M-15_ variants in *Salmonella* species in Nigeria. The continued existence of cefotaximase (CTX-M)-producing *Salmonella* within our environment calls for the prudent use of cephalosporins.

## Background

*Salmonella* infections are recognised as a global public health challenge and are one of the most common causes of human gastroenteritis worldwide, sometimes with negative clinical outcomes especially among vulnerable individuals [1–3]. Invasive salmonellosis is presented in approximately 6% of patients with a clinical prognosis of diarrhoeal enterocolitis. Invasive non-typhoidal *Salmonella* (iNTS) is endemic especially in sub-Saharan Africa occurring in 79% of the estimated 535, 000 global cases of iNTS infection and 85% of the estimated 77, 500 death globally giving a 15.8% case fatality ratio [4, 5].

*Salmonella*-associated infections are treated with ampicillin, chloramphenicol, and trimethoprim/sulfamethoxazole in the 1980s. However, the emergence of resistance clones to these antimicrobials resulted in the use of other antibiotic classes. Currently, several classes of antibiotics are available such as 3rd and 4th generation cephalosporin (cefpodoxime, ceftriaxone, ceftazidime, cefotaxime), fluoroquinolones (ciprofloxacin, moxifloxacin, levofloxacin) and carbapenems (ertapenem, doripenem, imipenem) which are essential in the treatment of salmonellosis with good therapeutic potentials. However, the misuse of these antibiotics classes in agricultural, animal and human medicine has been attributed to the emergence, persistence and eventual spread of resistant strains [6]. The continuous spread of multidrug-resistant bacterial pathogens has been attributed to prolonged hospitalisation causing increased morbidity and mortality [7].

*Salmonella* is increasingly becoming resistant from an estimated rate of 20–30% in the 1990s and increasing to over 30% a decade later [8]. There has been a global increase in the number of bacteria exhibiting resistance to third and fourth-generation cephalosporin, most times due to the production of extended-spectrum β-lactamases (ESBLs) and AmpC-type β-lactamases. Cefotaximase (CTX-M) type of ESBLs remains the widely distributed ESBL enzymes globally [9]. The prevalence rate of CTX-M-type β-lactamases differs among members of the family *Enterobacteriaceae* [10]. The earliest CTX-M-1 clone was detected in Germany in the late 1980s [11], consequently, an explosion of CTX-M-type β-lactamases was witnessed in the twenty-first century, with a report of CTX-M-2, 3, 4, 5, 6, 7, 9, 15 in South America, the Mediterranean and within the European countries [12].

The CTX-M-type β-lactamases belong to a novel Ambler molecular class A ESBL and are mostly plasmid-mediated with a preference for hydrolysing cefotaxime [13] with *bla*_CTX-M_-_15_ being most prevalent [14]. In Nigeria, the first _CTX-M-1_ clone was reported in 2015 [15]. Incidentally, only *bla*_CTX-M_-_1_, and *bla*_CTX-M_-_3_ clones have been documented by separate studies in *Salmonella enterica* [15, 16]. So far, the report on CTX-M-9 and 15 have only been documented in *Escherichia coli* isolated from clinical and animal sources in Nigeria [17, 18]. Eguale et al. [19] reported *bla*_CTX-M_-_15_ in *Salmonella* from Ethiopia_._ Many other studies in Egypt have recorded both *bla*_CTX-M-9_ and *bla*_CTX-M-15_ in *E. coli* [20, 21]. The distribution of CTX-M type of ESBLs among *Salmonella* species is becoming a serious challenge to global health. The continued dissemination of this resistant clone among members of the family *Enterobacteriales* in Nigeria is also becoming worrisome. There is a paucity of reports on the CTX-M type of ESBLs among *Salmonella* serovars in Nigeria, especially from clinical sources. This study aimed to investigate the prevalence of *Salmonella* in febrile and diarrhoeal patients, their antibiotic resistance, and the carriage of *bla*_CTX-M-9_ and *bla*_CTX-M-15_ ESBLs genes using real-time polymerase chain reaction (RT-PCR) in Lagos, Nigeria.

## Methods

### Ethics clearance

Ethical approvals from the Human Research and Ethics Committee of Lagos State University Teaching Hospital with reference number LREC/06/10/1012 and the Lagos State Health Service Commission with reference number LSHSC/2222/VOL.VC/352 were obtained prior to patients’ enrolment in accordance with 1964 Helsinki declaration and its later amendments or comparable ethical standards. The written informed consent was obtained from all subjects and/or their legal guardians.

### Study population, case definition and sample size

In this cross-sectional study, a total of 508 in-patients and out-patients who sought treatment at some public health care facilities in Lagos State, Nigeria, were recruited from April 2021 to August 2021. The patient’s history and demography were obtained. Two categories of patients were recruited for this study: category A includes patients with febrile illness. Patients with febrile illness were defined as a patient whose body temperature is greater than (≥ 37.5 °C) in the last 3 days with one or more of the following symptoms: abdominal pain, loose stool, headache, vomiting, and/or nausea, loss of appetite for up to 5 days. Category B includes patients with the diarrhoeal disease and was defined as a patient who had been diagnosed by a clinician with persistent loose stool for up to 72 h or more. The sample size was estimated using the formula for estimating the minimum sample for descriptive studies: *N* = *Z*^2^ *p* (1–*p*)**/***d*^2^, where, *N* = sample size, *Z* = 1.96 (standard error) at 95% confidence interval, *p* = prevalence of the disease as reported by Smith et al. [22] = 74%, and *d* = level of precision at 5% (0.05). Therefore, *N* = 295649536. Approximately 296 samples per 10,000 population. However, five hundred and eight (508) samples were collected to make the inference valid.

### Collection and processing of samples

Blood samples (10 ml from adults and 5 ml from children 1–15 yrs) were aseptically obtained and inoculated into blood culture bottles containing brain heart infusion (BHI) broth (LAB M, Lancashire, UK) and were gently mixed. The bottles were incubated at 37 °C aerobically for 7 days. Turbid bottles were sub-cultured on xylose lysine agar (XLD) (HIMEDIA, Mumbai, India), MacConkey agar (HIMEDIA, Mumbai, India), and *Salmonella–Shigella* Agar (SSA) (HIMEDIA, Mumbai, India) and were further incubated for 18–24 h at 37 °C. Non-turbid blood culture bottles were also sub-cultured and repeated daily from the BHI broth for up to 7 days, after which the blood-broth suspension was finally discarded. A loop full of stool samples was inoculated into 10 ml of alkaline peptone water (HIMEDIA, Mumbai, India) for pre-enrichment. The suspension was incubated for 18 h at 37 °C. One ml of the bacterial suspension was dispensed into 9 ml of Selenite F broth (HIMEDIA, Mumbai, India), and the suspension was incubated for at 37 °C for 18 to 24 h. After incubation, a loop full of the bacteria suspension was inoculated into MacConkey agar (HIMEDIA, Mumbai, India), blood agar (LAB M, Lancashire, UK), bismuth sulphite agar (BSA) (LAB M, Lancashire, UK), *Salmonella–Shigella* agar (SSA) (HIMEDIA, Mumbai, India), and xylose lysine agar (XLD) (HIMEDIA, Mumbai, India). The media were incubated at 37 °C for 18 to 24 h.

### Bacterial identification

Bacteria identification was carried out using the analytical profile index (API) 20E identification system (Institut Mérieux, Marcy l'Etoile, France) according to the manufacturer’s instructions. The results of several biochemical parameters were used to generate a profile index code. The profile index code generated was used for the identification of all the isolates in the API-WEB database (https://apiweb.biomerieux.com).

### Antimicrobial susceptibility testing (AST)

The in vitro antimicrobial susceptibilities of all identified *Salmonella* isolates to the commonly prescribed antibiotics in Nigeria were investigated by disk diffusion method, as described by the Clinical and Laboratory Standard Institute (CLSI) guidelines [23]. The following antibiotic discs were used: cefpodoxime (CPD) (30 µg), cefoxitin (FOX) (30 µg), cefotaxime (CTX) (30 µg), ceftazidime (CAZ) 30 µg, ampicillin (AMP) 10 µg, ciprofloxacin (CPR) 5 µg, augmentin (AUG) 30 µg, ofloxacin (OFL) 5 µg, imipenem (IMP) 10 µg, ertapenem (ETR) 10 µg, doripenem (DOR) 10 µg, amikacin 30 µg (AMK), and gentamycin (GEN) 10 µg (Oxoid, Hampshire, UK). Each of the antibiotic’s discs was placed aseptically on Mueller Hinton agar (HIMEDIA, Mumbai, India) plates that were previously inoculated with the *Salmonella* suspension equal to 0.5 McFarland standard and the plates were incubated aerobically at 37 °C for 18–24 h. The diameter of the zones of inhibitions was measured in millimetres and compared with a zone interpretation chart of CLSI [23]. *E. coli* ATCC® 25922™ was used as a control.

### Extended-spectrum β-lactamase (ESBL) assay

All *Salmonella* isolates with reduced susceptibility and/or resistance to 3rd generation cephalosporin (3GCs) were screened for ESBL production phenotypically using the double disk synergy test (DDST) method as previously described [16]. *Escherichia coli* ATCC® 25922™ was used as negative control while *Klebsiella pneumoniae* ATCC® 700603™ was used as a positive control.

### DNA extraction from bacterial isolate

The DNA extraction was carried out using a QIAamp DNA mini kit (Qiagen, Hilden, Germany) according to the manufacturer's instructions as described by Akinyemi et al. [16].

### RT-PCR

The RT-PCR Rotor gene Q 2plex (Qiagen, Hilden, Germany) was used for the amplification and detection of STY0313/SPA2475/t2576 and STY0316/t2574 for *Salmonella* Typhi and *Salmonella* Paratyphi A. *Salmonella* virulence genes *invA*, *stn*, and antibiotics resistance genes *bla*_CTX-M-9_, *bla*_CTX-M-15_, and *qnrB19* were screened using the TaqMan technology. The following previously published oligonucleotides sequences specific for detection of *invA*, *stn*, *bla*_CTX-M-9_, *bla*_CTX-M-15_, and *qnrB19* were used [16, 24–27] (Table 1). The qPCR assays were performed in a Qiagen rotor gene Q 2plex thermocycler (Qiagen, Hilden, Germany) with 72 well reaction tubes, closed with 4-Cap Strips. The reaction was performed by dispensing 12.5 μl of 2 × SYBR®Green Taq PCR master mix (Qiagen, Hilden, Germany) into 0.1 ml PCR tubes (Eppendorf, Germany). Then, 1.5 μl of both forward and reverse primers (Inqaba Biotec West Africa, Nigeria) and 10.5 μl of nucleic acid-free water (Qiagen, Hilden, Germany) were added. Finally, 1.5 μl of bacterial DNA (approximately 10^4^ copies of gDNA) was added to the mixture and amplified using the following protocol: hold temperature of 95 °C for 3 min, 40 cycles of denaturation at 94 °C for 1 min, annealing 64 °C for 30 s and extension at 72 °C for 30 s [16]. The threshold limit setting was performed in automatic mode. A “no template” control (NTC) was included in each reaction. Only signals generated in SYBR®Green qPCR analysis that displayed amplification above the threshold level were considered positive. While those signals with no *C*_*t*_ value obtained were considered negative.

**Table 1:** Primers with nucleotide sequence use in the study

Primers	Oligonucleotide sequence	References
*InvA-*F	5’- ACCACGCTCTTTCGTCTGG-3’	[16]
*InvA-*R	5’- GAACTGACTACGTAGACGCTC-3’	
_*CTX-M*-9-_F	5'-TTACAAACCGTCGGTGACGA-3'	[24]
_*CTX-M*-9_-R	5-GT GAC AAA GAG AGT GCA ACG G-3	
_*CTX-M*-15-_F	5'-GGTTAAAAAATCACTGCGTC-3'	[25]
_*CTX-M*-15-_R	5'-TTACAAACCGTCGGTGACGA-3'	
*qnr*B19-F	5'-CRATGTGCAGYACCAGTAA-3'	[25]
*qnr*B19-R	5'-CGCRATATCRTTGGTGGTG-3'	
*Stn-*F	5'- ACCACGCTCTTTCGTCTGG-3'	[26]
*Stn-*R	5'- GAACTGACTACGTAGACGCTC-3'	
STY0313/SPA2475/t2576-F	5-CTTGACGTACCGGTAGAGAT ATACTGGCT-3	[27]
STY0316/t2574-R	5-CTTGACGTACCGGTAGAGAT ATACTGGCT-3	

### Statistical analysis

These data generated were entered into Microsoft Excel spreadsheet 2010 and were analysed using Statistical Package for Social Sciences (SPSS) Windows (Version 15.2; Chicago, IL) and Epi Info (Version 6.0, USD, Stone Mountain, GA). Prevalence of *Salmonella* and its association with demographic determinants among febrile and diarrhoeagenic patients in selected health care facilities were determined. The prevalence of salmonellosis was calculated as the number of positive cultures for *Salmonella* divided by the total number of samples collected. The data were interpreted using the Chi-square test of independence, while the strength of association was determined by estimating the odds ratio (OR). The statistical test was considered significant if the *P*-value was ≤ 0.05. A correlation analysis was also performed to determine the association of virulence genes and antimicrobial resistance genes belonging to the same antimicrobial class or different classes among the investigated *Salmonella* isolates. Binary data (0) indicating absent and (1) indicating presence of virulence and resistance genes were imported into an R software (version 4.1.3 and RStudio IDE. https://www.r-project.org; https://www.rstudio.com. Accessed, 3 September 2022). The correlations were then calculated with package “ggcorrplot” using the function cor.mtest” at a significance of *P* < 0.05.

## Results

### *Salmonella* and other bacterial isolates

In total, 229 bacterial pathogens were isolated from 508 samples obtained from febrile and diarrhoeagenic patients. Twenty-four *Salmonella* species were identified from the total sample. Other non-*Salmonella* bacterial pathogen isolated were as follows: *Proteus vulgaris* (12.2%), *Proteus mirabilis* (9.6%), *E. coli* (9.3%), *Klebsiella oxytoca* (4.3%), *Klebsiella pneumoniae* (3.3%), *Edwardsiella* species (2.0%), *Enterobacter* species (1.4%), *Citrobacter* species (0.6%), *E. coli* 0157H7 (0.4%), and *Staphylococcus* species (0.4%).

### Demographic distribution of *Salmonella* isolates

The proportion of *Salmonella* species was 6.3% (15/240) in males and 3.4% (9/268) in females. Out of the 24 *Salmonella* species detected, 15/24 representing 62.5%, were isolated from children between the ages of 1–10 years, with a mean age of 4.7 years. Also, 25.0% (6/24) of the total *Salmonella* isolates were detected from patients between the ages of 11–20 years, with a mean age of 14.3 years. Meanwhile, the remaining 12.5% (3/24) was isolated from patients within the age bracket 21–30 years with a mean age of 24.7 years (Tables 2 and 3). The prevalence of *Salmonella*-associated bacteraemia stood at 3.9% (15/378), while *Salmonella*-associated gastroenteritis was 6.9% (9/130). The relationship between *Salmonella* detections, gender, age distribution, clinical prognosis, and study centres reveals a significant association with a *P*-value < 0.0001, odd ratio (OR) ranging from (0.0180–0.1316), at 95% CL (Table 3).

**Table 2:** Distribution of *Salmonella* and other bacterial pathogens isolated from febrile and diarrhoeagenic patients in Lagos, Nigeria

Variables	Category	Number tested	Number positive culture	*Salmonella* species	*E. coli*	*E. coli* 0157H7	*P. mirabilis*	*K. pneumoniae*	*Citrobacter sp*	*P. vulgaris*	*Staphylococcus sp*	*Klebsiella oxytoca*	*Enterobacter sp*	*Edwardsiella sp*
Gender	Male	240	117	15 (6.3)	17 (7.1)	0 (0)	21 (8.8)	8 (3.33)	1 (4.6)	32 (13.33)	2 (0.83)	10 (4.2)	5 (2.08)	6 (2.5)
	Female	268	128	9 (3.4)	30 (11.2)	2 (0.7)	28 (10.5)	9 (3.4)	2 (0.8)	30 (11.2)	(0.90)	12 (4.5)	2 (0.8)	4 (1.5)
Age	1–10 years	129	77	15 (11.6)	22 (17.1)	2 (1.6)	12 (9.3)	2 (1.6)	1 (0.78)	20 (15.5)	1 (0.78)	6 (4.7)	2 (1.6)	4 (3.1)
	11–20 years	104	50	6 (5.8)	10 (9.6)	0 (0)	10 (9.6)	4 (3.8)	0 (0)	14 (13.5)	0 (0)	6 (5.8)	2 (1.9)	2 (1.9)
	21–30 years	78	32	3 (3.8)	5 (6.4)	0 (0)	8 (10.3)	2 (2.6)	1 (1.3)	12 (15.4)	0 (0)	1(1.3)	1 (1.3)	1 (1.3)
	31–40 years	63	23	0 (0)	3 (4.8)	0 (0)	4 (6.3)	3 (4.8)	0 (0)	8 (12.7)	0 (0)	4 (6.3)	0 (0)	1 (1.6)
	41–50 years	42	14	0 (0)	2 (4.8)	0 (0)	3 (7.1)	3 (7.1)	0 (0)	2 (4.8)	0 (0)	2 (4.8)	1 (0.2)	1 (0.2)
	51 years and above	31	12	0 (0)	2 (6.5)	0 (0)	3 (9.7)	1 (3.2)	1 (3.2)	2 (6.5)	0 (0)	1 (3.2)	1 (3.2)	1 (3.2)
	No age (adults)	61	21	0 (0)	3 (4.9)	0 (0)	9 (14.8)	2 (3.3)	0 (0)	4 (6.6)	1 (1.6)	2 (3.3)	0 (0)	0 (0)
Clinical prognosis/sample type	Febrile illness (blood)	378	147	15 (3.96)	0 (0)	0 (0)	41 (10.8)	15 (3.96	1 (0.3)	51 (13.5)	2 (0.5)	19 (5.02)	5 (1.3)	8 (2.1)
	Diarrhoeal disease (stool)	130	82	9 (6.9)	47 (36.2)	2 (1.5)	8 (6.2)	2 (1.5)	2 (1.5)	11 (8.5)	0 (0)	3 (2.3)	2 (1.5)	2 (1.5)
Study centres	Lagos State University teaching Hospital (blood abd stool)	170	83	3 (1.8)	27 (15.9)	0 (0)	17 ((10)	8 (4.7)	1 (0.6)	17 (10)	1 (0.6)	3 (1.8)	4 (2.4)	4 (2.4)
	Randle General Hospital (blood)	140	62	12 (8.6)	0 (0)	0 (0)	13 (9.3)	1 (0.7)	1 (0.7)	27 (19.3)	0 (0)	12 (8.6)	0 (0)	4 (2.9)
	Life font Hospital (blood)	70	24	3 (4.3)	0 (0)	0 (0)	6 (8.6)	3 (4.3)	0 (0)	9 (12.9)	0 (0)	4 (5.7)	0 (0)	1 (1.4)
	Alimosho General Hospital (blood and stool)	128	60	6 (4.7)	20 (15.6)	2 (1.6)	13 (10.2)	5 (3.9)	1 (0.8)	9 (7.0)	1 (0.8)	3 (2.3)	3 (2.3)	1 (0.8)
Total		508	245	24 (4.7)	47 (9.3)	2 (0.4)	49 (9.6)	17 (3.3)	3 (0.6)	62 (12.2)	2 (0.4)	22 (4.3)	7 (1.4)	10 (1.96)

**Table 3:** Prevalence of *Salmonella* and its association with demographic determinant among febrile and diarrhoeagenic patients in selected health care facilities

Variable	Category	No. tested	No. of + ve culture	No. of *Salmonella* Detected	Occurrence (%) (n-24)	Prevalence (%)	Odds ratio	95% CI	*z* statistic	*P*-value
Gender	Male	240	107	15	62.5	6.25	0.0667	0.0384–0.1158	9.608	< 0.0001
	Female	268	122	9	37.5	3.4	0.0347	0.0175–0.0690	9.601	< 0.0001
Age distribution	1–10	129	77	15	62.5	11.6	0.1316	0.0729–0.2376	6.725	< 0.0001
	11–20	104	50	6	25	5.8	0.0612	0.0257–0.1458	6.308	< 0.0001
	21–30	78	32	3	12.5	3.8	0.0400	0.0121–0.1323	5.275	< 0.0001
Diagnosis	Febrile illness	378	147	15	62.5	3.9	0.0413	0.0242–0.0706	11.657	< 0.0001
	Diarrhoeal disease	130	82	9	37.5	6.9	0.0744	0.0362–0.1527	7.079	< 0.0001
Study centre	LASUTH	170	83	3	12.5	1.8	0.0180	0.0056–0.0574	6.783	< 0.0001
	RGH	140	62	12	50	8.6	0.0938	0.0496–0.1772	7.290	< 0.0001
	LFMC	70	24	3	12.5	4.3	0.0448	0.0134–0.1491	5.060	< 0.0001
	AGH	128	60	6	25	10.0	0.0492	0.0209–0.1157	6.902	< 0.0001

*LASUTH* Lagos State University Teaching Hospital, *RGH* Randle General Hospital, *LFMC* Life-Font Medical Centre, *AGH* Alimosho General Hospital, *ND* not determined, *NA* not determined

### Antibiotic susceptibility and pattern of resistance

The result of the in vitro antimicrobial susceptibility testing showed some level of susceptibility to imipenem (75.0%), ciprofloxacin (62.5%), gentamycin (62.5%) and ofloxacin (50.0%), while all the isolates developed resistance (100.0%) to ceftazidime, cefotaxime, cefoxitin, augmentin, ampicillin, ertapenem, and doripenem. However, 75.0% of the isolates showed resistance to cefpodoxime. Resistance to ofloxacin and amikacin was 50.0% each. All *Salmonella* isolates were multiple drug resistant (MDR). MDR was defined as resistance to at least one agent in three or more antimicrobial categories (Table 4). All the isolates developed resistance to between 7 and 13 antibiotics, with 5 patterns of resistance observed. Six isolates each have pattern CAZ-CPD-CTX-FOX-AUG-AMP-GEN-AMK-CPR-OFL-IMP-ETR-DOR, and CAZ-CPD-CTX-FOX-AUG-AMP-ETR-DOR, five isolates with pattern CAZ-CTX-FOX-AUG-AMP-ETR-DOR, while three isolates each have CAZ-CPD-CTX-FOX-AUG-AMP-GEN-AMK-CPR-OFL-ETR-DOR and CAZ-CPD-CTX-FOX-AUG-AMP-AMK-OFL-IMP-ETR-DOR as their pattern of resistance and one isolate with resistant pattern CAZ-CPD-CTX-AUG-AMP-ETR-DOR (Table 5). High multiple antibiotic resistance (MAR) index (ranging from 0.5 to 1.0) was observed for all the *Salmonella* isolates. All the *Salmonella* isolates from this study were negative when screened for *Salmonella* Typhi and *Salmonella* Paratyphi A gene using STY0313/SPA2475/t2576-F and STY0316/t2574-R primer pairs (Table 6).

**Table 4:** Antibiogram of non-typhoidal *Salmonella* isolates from clinical samples by disc diffusion method

Class of antibiotics	Antimicrobial agent	Resistant %	Susceptible %
Cephalosporin	Ceftazidime (CAZ) (30 µg)	24 (100.0)	0 (0.0)
	Cefpodoxime (CPD) (30 µg)	18 (75.0)	6 (25.0)
	Cefotaxime (CTX) (30 µg)	24 (100.0)	0 (0.0)
	Cefoxitin (FOX) (30 µg)	24 (100.0)	0 (0.0)
Penicillin	Augmentin (AUG) 30 µg	24 (100.0)	0 (0.0)
	Ampicillin (AMP) 10 µg	24 (100.0)	0 (0.0)
Fluoroquinolone	Ciprofloxacin (CPR) 5 µg	9 (37.5)	15 (62.5)
	Ofloxacin (OFL) 5 µg	12 (50.0)	12 (50.0)
Carbapenem	Doripenem (DOR) 10 µg	24 (100.0)	0 (0.0)
	Ertapenem (ETR) 10 µg	24 (100.0)	0 (0.0)
	Imipenem (IMP) 10 µg	6 (25.0)	18 (75.0)
Aminoglycoside	Gentamycin (GEN) 10 µg	9 (37.5)	15 (62.5)*
	Amikacin (AMK) 30 µg	12 (50.0)	12 (50.0)*

*In vitro susceptibility does not mean the antibiotics are effective in vivo and should be considered as resistant

**Table 5:** Antibiotics resistant pattern of MDR-*Salmonella* species isolated from febrile and diarrhoeal patients

No. of profile	Antibiotics resistant pattern	No. of antibiotics resisted	*Salmonella *species
1	CAZ-CPD-CTX-FOX-AUG-AMP-GEN-AMK-CPR-OFL-IMP-ETR-DOR	13	6
2	CAZ-CPD-CTX-FOX-AUG-AMP-GEN-AMK-CPR-OFL-ETR-DOR	12	3
3	CAZ-CPD-CTX-FOX-AUG-AMP-AMK-OFL-IMP-ETR-DOR	11	3
4	CAZ-CPD-CTX-FOX-AUG-AMP-ETR-DOR	8	6
5	CAZ-CTX-FOX-AUG-AMP-ETR-DOR	7	5
6	CAZ-CPD-CTX-AUG-AMP-ETR-DOR	7	1

^*^Ceftazidime (CAZ), cefpodoxime (CPD), cefotaxime (CTX), cefoxitin (FOX), augmentin (AUG), ampicillin (AMP), ciprofloxacin (CPR), ofloxacin (OFL), doripenem (DOR), ertapenem (ETR), imipenem (IMP), gentamycin (GEN)

**Table 6:** Characteristics of *invA*, *bla*_CTX-M-9_ and *bla*_CTX-M-15_ gene-harbouring *Salmonella* isolates

Isolate ID	Sample type	Age	Sex	Clinical prognosis	Resistance pattern	No of antibiotics resisted (%)	MAR Index	S. Typhi/S. Paratyphi A	Virulence gene		Antibiotics resistant gene		
									*inv*A (*C*_*t*_ value)	*Stn*	*qnrB*19	*bla*_CTX-M-9_ (*C*_*t*_ value)	*bla*_CTX-M-15_ (*C*_*t*_ value)
S101	Stool	6	M	Diarrheal disease	CAZ-CPD-CTX-FOX-AUG-AMP-GEN-AMK-CPR-OFL-IMP-ETR-DOR	13 (100.0)	1.0	–	+ (20.4)	–	–	+ (37.15)	–
S102	Blood	8	M	Febrile illness	CAZ-CPD-CTX-FOX-AUG-AMP-GEN-AMK-CPR-OFL-IMP-ETR-DOR	13 (100.0)	1.0	–	+ (23.30)	–	–	–	+ (29.29)
S103	Blood	4	M	Febrile illness	CAZ-CPD-CTX-FOX-AUG-AMP-GEN-AMK-CPR-OFL-ETR-DOR	12 (92.3)	0.9	–	+ (23.59)	–	–	+ (33.14)	–
S104	Blood	13	F	Febrile illness	CAZ-CPD-CTX-FOX-AUG-AMP-AMK-OFL-IMP-ETR-DOR	11 (84.6)	0.8	–	+ (39.53)	–	–	–	–
S105	Blood	20	F	Febrile illness	CAZ-CPD-CTX-FOX-AUG-AMP-ETR-DOR	8 (61.5)	0.6	–	-	–	–	+ (25.94)	+ (38.33)
S106	Stool	2	F	Diarrheal disease	CAZ-CPD-CTX-FOX-AUG-AMP-ETR-DOR	8 (61.5)	0.6	–	-	–	–	–	–
S107	Stool	5	M	Diarrheal disease	CAZ-CTX-FOX-AUG-AMP-ETR-DOR	7 (53.6)	0.5	–	+ (27.15)	–	–	+ (30.62)	–
S108	Blood	28	M	Febrile illness	CAZ-CTX-FOX-AUG-AMP-ETR-DOR	7 (53.6)	0.5	–	+ (27.60)	–	–	+ (29.60)	–
S109	Blood	8	F	Febrile illness	CAZ-CPD-CTX-FOX-AUG-AMP-ETR-DOR	8 (61.5)	0.6	–	+ (39.53)	–	–	+ (25.40)	+ (30.29)
S110	Blood	2	F	Febrile illness	CAZ-CTX-FOX-AUG-AMP-ETR-DOR	7 (53.6)	0.5	–	+ (31.60)	–	–	+ (21.70)	–
S111	Blood	1	M	Febrile illness	CAZ-CPD-CTX-FOX-AUG-AMP-GEN-AMK-CPR-OFL-ETR-DOR	12 (92.3)	0.9	–	-	–	–	+ (29.72)	–
S112	Blood	15	M	Febrile illness	CAZ-CPD-CTX-FOX-AUG-AMP-AMK-OFL-IMP-ETR-DOR	11 (84.6)	0.8	–	+ (23.30)	–	–	–	–
S113	Blood	20	M	Febrile illness	CAZ-CPD-CTX-FOX-AUG-AMP-ETR-DOR	8 (61.5)	0.6	–	+ (22.21)	–	–	–	–
S114	Stool	7	F	Diarrheal disease	CAZ-CPD-CTX-FOX-AUG-AMP-GEN-AMK-CPR-OFL-IMP-ETR-DOR	13 (100.0)	1.0	–	+ (28.09)	–	–	–	+ (20.31)
S115	Stool	4	M	Diarrheal disease	CAZ-CPD-CTX-FOX-AUG-AMP-GEN-AMK-CPR-OFL-IMP-ETR-DOR	13 (100.0)	1.0	–	-	–	–	+ (30.20)	–
S116	Stool	24	F	Diarrheal disease	CAZ-CTX-FOX-AUG-AMP-ETR-DOR	7 (53.6)	0.5	–	-	–	–	+ (33.64)	–
S117	Stool	5	F	Diarrheal disease	CAZ-CPD-CTX-FOX-AUG-AMP-GEN-AMK-CPR-OFL-IMP-ETR-DOR	13 (100.0)	1.0	–	-	–	–	+ (33.24)	–
S118	Stool	9	M	Diarrheal disease	CAZ-CPD-CTX-FOX-AUG-AMP-GEN-AMK-CPR-OFL-IMP-ETR-DOR	13 (100.0)	1.0	–	-	–	–	–	+ (29.29)
S119	Blood	2	M	Febrile illness	CAZ-CPD-CTX-FOX-AUG-AMP-ETR-DOR	8 (61.5)	0.6	–	+ (23.30)	–	–	+ (20.23)	+ (31.47)
S120	Blood	16	M	Febrile illness	CAZ-CPD-CTX-FOX-AUG-AMP-ETR-DOR	8 (61.5)	0.6	–	+ (27.15)	–	–	–	–
S121	Blood	19	F	Febrile illness	CAZ-CTX-FOX-AUG-AMP-ETR-DOR	7 (53.6)	0.5	–	+ (28.06)	–	–	+ (37.15)	–
S122	Blood	5	M	Febrile illness	CAZ-CPD-CTX-AUG-AMP-ETR-DOR	7 (53.6)	0.5	–	-	–	–	+ (25.94)	–
S123	Stool	7	M	Diarrheal disease	CAZ-CPD-CTX-FOX-AUG-AMP-GEN-AMK-CPR-OFL-ETR-DOR	12 (92.3)	0.9	–	+ (28.06)	–	–	+ (33.40)	–
S124	Blood	30	M	Febrile illness	CAZ-CPD-CTX-FOX-AUG-AMP-AMK-OFL-IMP-ETR-DOR	11 (84.6)	0.8	–	+ (31.60)	–	–	+ (29.20)	–

^*^Ceftazidime (CAZ), cefpodoxime (CPD), cefotaxime (CTX), cefoxitin (FOX), augmentin (AUG), ampicillin (AMP), ciprofloxacin (CPR), ofloxacin (OFL), doripenem (DOR), ertapenem (ETR), imipenem (IMP), gentamycin (GEN)

### Virulence and CTX-M genes

Sixteen of the isolates (66.7%) harboured the invasive (*inv*A) gene, while none of the isolates harboured the enterotoxin (*stn*) gene. In total, 79.2% (19/24) of the *Salmonella* isolates harboured the *bla*_CTX-M_ variant including 52.0% (13/24) *bla*_CTX-M-9_ and 12.5% (3/24) *bla*_CTX-M-15_, respectively. Six *Salmonella* isolates from diarrhoeal patients and seven *Salmonella* isolates from patients with febrile illness harboured *bla*_CTX-M-9_ only_._ Two *Salmonella* isolates from febrile patients and one from diarrhoeal patients harboured *bla*_CTX-M-15_ only, respectively. Of note was the co-existence of *bla*_CTX-M-9_ and *bla*_CTX-M-15_ in 3 (12.5%) *Salmonella* isolates from febrile patients with similar resistance pattern CAZ-CPD-CTX-FOX-AUG-AMP-ETR-DOR. All the isolates were negative for the plasmid-mediated quinolone resistance gene *qnr*B19 (Table 6).

### Correlation analysis

**Fig. 1:**
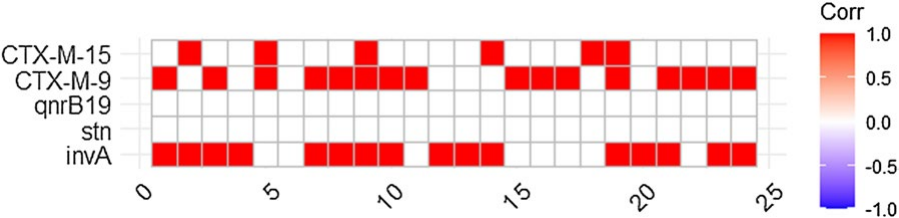
Correlation analysis determining the associations between virulence and resistance genes among non-typhoidal *Salmonella* isolates from hospitalised patients in Lagos, Nigeria. The red boxes indicate positive association, while the strength of colour corresponds to the numerical value of the correlation coefficient (*r*). Significance was calculated at *P* < 0.05, and boxes with non-significant correlations were left blank

The result of the correlation analysis revealed a significant association between the *inv*A gene and *bla*_CTX-M-9_ and a strong association between *bla*_CTM-9_ and *bla*_CTX-M-15._ Significance was calculated at *P* < 0.05, and boxes with non-significant correlations were left blank as such genes were not detected (Fig. 1).

## Discussion

*Salmonella*-associated infection caused by the genus *Salmonella* remains a public health challenge in Africa. *Salmonella* has been associated with an increasing number of reported cases of community-acquired bloodstream infections, self-limiting gastroenteritis to more severe infections resulting in a high rate of morbidity and mortality [16]. Fluoroquinolone and extended-spectrum cephalosporin are used as first-line antibiotics for the treatment of salmonellosis. However, treatment failure due to resistance to these classes of antibiotics has resulted in prolonged hospitalisation with negative clinical outcomes [7]. In this study, 24 *Salmonella* species were detected from 508 samples with an overall prevalence of 4.7%. The prevalence of *Salmonella*-associated bacteraemia was 3.9%, while *Salmonella*-associated gastroenteritis was 6.9%. This result was similar to 3.9% prevalence of *Salmonella*-associated bacteraemia reported in Nigeria by Akinyemi et al. [16], but at variance with the 1.9% recorded by the same author in a separate study [28]. All the *Salmonella* species detected in this study were non-typhoidal *Salmonella* because none of the primer pairs used for the detection of *Salmonella* Typhi and *Salmonella* Paratyphi A yielded Ct value. The Prevalence of *Salmonella*-associated gastroenteritis (SAG) in this study was 6.9%. The result was lower when compared to the results of similar studies; 9.6%, 16.3% and 16.6% that were conducted in different periods in 2021, 2018 and 2007 in Lagos, Nigeria [16, 29, 30], respectively. However, this study was consistent with the 6.2% (59/957) reported in Ethiopia [31]. Conversely, lower prevalence of 1.3% and 2.7% were reported in separate studies in Ethiopia [32, 33], 3.0% in Kenya [34] and 3.5% in Egypt [35] compared to 6.9% SAG reported in this study. Also, the 6.9% SAG recorded in this study was lower when compared to the 13.80% in Tanzania [36] 17.86% in Thailand [37] and 20.39% in China [38]. The observed variations in the prevalence of *Salmonella* infection in this study when compared to other studies, could be attributed to various factors such as sample size, gender, and age distribution, location of sampling and seasonal variation [36].

Fifteen out of the 20 (15/24) *Salmonella* species detected representing 62.5% were isolated from children between the ages of 1–10 years, with a mean age of 4.7 years, standard deviation (SD) ± 2.859, margin of error (ME) ± 0.2517 (4.7364 ± 0.494 (± 10.42%) at 95% confidence level. The prevalence of *Salmonella* infection in this study was higher among children < 10 years when compared to other age groups. Children have been identified as a vulnerable population group at risk of *Salmonella* infection due to underdeveloped immune system and exhibition of poor hygienic habits [38]. Report on high prevalence of *Salmonella* infection in children is not uncommon. Several studies have documented a high prevalence of *Salmonella* in children in different countries such as Nigeria [16, 39], China [38, 40], and Italy [41]. There were variations in the observed clinical presentations of the patients in this study and intravenous saline (0.8%) was administered in those patients with severe dehydration. The result of the in vitro antimicrobial susceptibility testing showed that all the 24 *Salmonella enterica* detected were MDR as they were resistant to three or more classes of antibiotics. Notably, 100% resistance to all the following β-lactam antibiotics (ceftazidime, cefotaxime, cefoxitin, augmentin and ampicillin) and carbapenems (ertapenem and doripenem) were observed in all the *Salmonella* species, while 75% (18/24) *Salmonella* isolates showed resistance to cefpodoxime. Regrettably, in Nigeria, similar level of resistance was reported by other studies [16, 42, 43]. Studies conducted elsewhere recorded similar resistance in *Salmonella* species. In Ethiopia, 88.9% of all the isolates were resistant to ampicillin [28], 88% in Bangladesh [40] and 74% resistance to cefotaxime was reported by Harakeh et al. [44] in Lebanon. High-level resistance to β-lactam antibiotics was also reported in Burkina Faso [45], Canada [46], and the USA [47]. The high level of resistance among *Salmonella* to β-lactam antibiotics and carbapenems as revealed in this study is worrisome and calls for urgent attention. The differences in the percentage of resistance from different countries may be due to different risk factors such as sickle cell disease, HIV, malnutrition, tuberculosis and other respiratory infections, all of which are conditions known to be associated with frequent antibiotic use [39]. Reduced susceptibility to imipenem (25.0%), ciprofloxacin (37.5%), ofloxacin (50.0%), gentamycin (37.5%), and amikacin (50.0%) was recorded in this study. Reduced susceptibility to fluoroquinolones is a serious public health concern, as this class of antibiotics remains among the antimicrobials of choice for the treatment of invasive and systemic salmonellosis in human medicine and animals’ husbandry [48, 49]. This study revealed high multiple antibiotic resistance (MAR) index (ranging from 0.5 to 1.0) in all the *Salmonella* isolates with five heterogeneous patterns of resistance. *Salmonella* isolates have been reported in Nigeria to display similar resistant phenotype [16, 42]. Studies elsewhere have also shown to express similar resistance phenotypes consistent with this study. For instance, Asfaw Ali et al. [50] reported that 11 out of the 43 *Salmonella* isolates exhibited the same resistance phenotype. Also, Eguale et al. [19] reported high MAR index with similar resistance pattern in *S*. Virchow, *S.* Typhimurium, *S*. Saint-Paul, *S*. Kentucky, *S*. Heidelberg and *S*. Concord in their study. The expression of similar resistance phenotype by different *Salmonella* serovars makes the resistance profile an unreliable typing tool. Despite the phenotypic expression of high-level resistance to β-lactam antibiotics, phenotypic expression of ESBL was not recorded in this study with the double disc synergy test used. The negative impact of the two enzyme groups (AmpC and ESBL) which have overlapping hydrolysis spectra and the presence of ESBL genes have been reported to affect the phenotypic expression of ESBL using the convectional screening methods [51]. It is noteworthy that, 79.2% (19/24) *Salmonella* isolates harboured *bla*_CTX_ genes. Thirteen of the *Salmonella* isolates carry *bla*_CTX-M-9_ genes only, while three of the *Salmonella* isolates harboured *bla*_CTX-M-15_ genes only. Both *bla*_CTX-M-9_ and *bla*_CTX-M-15_ were detected in *Salmonella* isolates from diarrhoeal patients and febrile patients with persistent pyrexia. Interestingly, co-existence of *bla*_CTX-M-9_ and *bla*_CTX-M-15_ was observed in three *Salmonella* spp isolated from febrile patients with resistance pattern CAZ-CPD-CTX-FOX-AUG-AMP-ETR-DOR. Detection of *bla*_CTX-M-15_ and *bla*_CTX-M-9_ from the clinical samples have been reported in Iran [52]. Ethiopia [19], India [9] Zambia [53], Kuwait [54], Japan [53], France and Senegal [25], and Germany [55]. Therefore, this study has demonstrated the potential dissemination of *bla*_CTX-M_ variants in *Salmonellae* from our environment with a prolonged hospital stay of patients and consequently resulting in treatment failure. Resistance to this class of antibiotic by *Salmonella* has generated global public health concern [56], and this is now a major concern in Nigeria as 50% of the patients harbouring *Salmonella* with _CTX-M_ genes in this study are children < 10 years of age. There have been successful global dissemination of _CTX-M_ genes, two of these most common variant bla_CTX-M-9_ and *bla*_CTX-M-15_ were detected in this study. Although, in Nigeria, *bla*_CTX-M-9_ and *bla*_CTX-M-15_ have been reported in *E. coli* isolated from stool samples of pregnant women [17] and from animal sources [18]. To the best of our knowledge, this is the first report of *bla*_CTX-M-9_ and *bla*_CTX-M-15_ in *Salmonella* isolated from clinical sources in Nigeria. In this study, out of the *bla*_CTX-M-9_ and *bla*_CTX-M-15_ -producing *Salmonella* strains detected, 50% (12/24) were simultaneously resistant to ciprofloxacin and ofloxacin in vitro. However, none of these isolates harboured plasmid-mediated quinolone-resistant gene *qnr*B19 genes. This gene if present would have enhance the development of quinolone resistance strains [57]. None of the *Salmonella* isolates from this study harboured the enterotoxin (*stn*) gene. The expression of this gene is responsible for *Salmonella*-associated gastroenteritis by inducing previously been reported in Nigeria [59] and Egypt [60]. However, the detection of invasive (*invA*) gene which is a common molecular target for *Salmonella-*specific detection was recorded in 66.7% (16/24) of *Salmonella* species investigated, an indication that qPCR investigation of *invA* gene is a suitable target for the confirmation of putative *Salmonella* isolates. The *invA* gene of *Salmonella* species is associated with invasion of host epithelial cell and it is located on the pathogenicity island 1 (SPI-1). The detection of this virulence gene in *Salmonella* as a diagnostic application had been reported by several authors with varying degree of sensitivity and specificity ranging from 55 to 100% [16, 61, 62].

## Conclusion

This study revealed prevalence of 4.7% *Salmonella*-associated infections, with 62.5% of the *Salmonella * isolates detected found in children ≤ 10 years. All *Salmonella* isolated exhibited high level of resistance to β-lactams antibiotics and carbapenems with reduced susceptibility to fluoroquinolone. The study revealed for the first time the carriage of *bla*_CTX-M-9_ and *bla*_CTX-M-15_ variants among the *Salmonella* species from Nigeria. Complete therapeutic failure of first-line antibiotics is eminent, if the policy-makers do not only ensure a complete barn on the over-the-counter-sale of antibiotics without doctor’s prescription, but also a routine follow-up programme for enforcement, to curb the spread of antibiotics resistance organisms.

## Data Availability

All data generated or analysed during this study are included here and are available from the corresponding author on reasonable request.

## Abbreviations

AST: Antibiotic susceptibility testing
CTX-M: Cefotaximase
ESBLs: Extended-spectrum β-lactamases
iNTS: Invasive non-typhoidal *Salmonella*
qPCR: Quantitative polymerase chain reaction
